# Boron–Silicon Alloy Nanoparticles as a Promising New Material in Lithium-Ion Battery Anodes

**DOI:** 10.1021/acsenergylett.4c00856

**Published:** 2024-05-02

**Authors:** Gregory F. Pach, Pashupati R. Adhikari, Joseph Quinn, Chongmin Wang, Avtar Singh, Ankit Verma, Andrew Colclasure, Jae Ho Kim, Glenn Teeter, Gabriel M. Veith, Nathan R. Neale, Gerard M. Carroll

**Affiliations:** † Chemistry and Nanoscience Center, National Renewable Energy Laboratory, 15013 Denver West Parkway, Golden, Colorado 80401, United States; ‡ Environmental Molecular Sciences Laboratory, Pacific Northwest National Laboratory, Richland, Washington 99342, United States; § Energy Conversion and Storage Systems Center, National Renewable Energy Laboratory, Golden, Colorado 80401, United States; ∥ Department of Nanoenergy Engineering, Pusan National University, Busan 46241, Republic of Korea; ⊥ Materials Sciences Center, National Renewable Energy Laboratory, 15013 Denver West Parkway, Golden, Colorado 80401, United States; # Chemical Sciences Division, Oak Ridge National Laboratory, Oak Ridge, Tennessee 37831, United States; ○ Renewable and Sustainable Energy Institute, University of Colorado Boulder, Boulder, Colorado 80309, United States

## Abstract

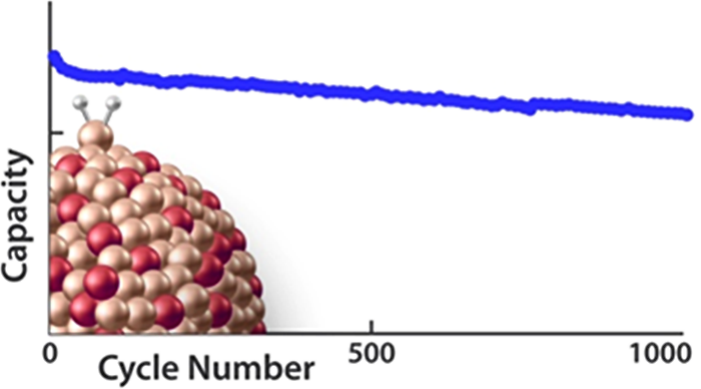

Silicon’s potential as a lithium-ion battery (LIB) anode is hindered by the reactivity of the lithium silicide (Li_*x*_Si) interface. This study introduces an innovative approach by alloying silicon with boron, creating boron/silicon (BSi) nanoparticles synthesized via plasma-enhanced chemical vapor deposition. These nanoparticles exhibit altered electronic structures as evidenced by optical, structural, and chemical analysis. Integrated into LIB anodes, BSi demonstrates outstanding cycle stability, surpassing 1000 lithiation and delithiation cycles with minimal capacity fade or impedance growth. Detailed electrochemical and microscopic characterization reveal very little SEI growth through 1000 cycles, which suggests that electrolyte degradation is virtually nonexistent. This unconventional strategy offers a promising avenue for high-performance LIB anodes with the potential for rapid scale-up, marking a significant advancement in silicon anode technology.

Silicon is envisioned to replace graphite as the next-generation anode active material in lithium-ion batteries (LIBs),^1^ but passivating the reactive lithium silicide (Li_*x*_Si) interface against parasitic chemistries has proven to be a formidable challenge.^2^ The initial lithiation and delithiation of a silicon anode produce an amorphous and poorly defined composite material at the surface of the silicon known as the solid electrolyte interphase (SEI). Ideally, the SEI would passivate the Li_*x*_Si surface, but this has not been the case. Irreversible chemistry related to Li^+^ and anion consumption as well as solvent reduction occur at each charge/discharge cycle which compound on each other resulting in rapid capacity fade and battery failure.^3^ Moreover, even without electrochemical lithiation and delithiation, Si-containing anodes spontaneously react with electrolyte under open-circuit conditions,^4,5^ which is known as calendar aging.^6^

Several strategies have been put forward to stabilize the Li_*x*_Si surface. Beyond exploring new electrolyte compositions, the most common strategy for reducing the parasitic chemistry of Li_*x*_Si is to bury the interface in a nonreactive material that does not inhibit lithiation and delithiation.^1,7−15^ This strategy has been explored for various carbons,^8,16−20^ polymers and oligomers,^21−23^ metal oxides,^24^ and many other materials. These buried interfaces have even been engineered to account for the 350% volume expansion upon lithiation—the most famous being the “yolk–shell”^25^ and “pomegranite”^26^ structures. Despite the tremendous volume of research and apparent successes in passivation, however, commercial LIB anodes for high energy density applications are largely composed of high-content graphite and low-content silicon composites, indicating that none of these solutions have bridged the technological gap between lab and commercial scale.

A far less explored but promising area of materials design is to change the composition of the silicon itself through alloying and doping.^27−29^ More specifically, doping with elements that affect the electronic energy levels and surface chemistry. Silicon, doped with aliovalent elements, is the lynchpin of the modern electronics industry. Through doping, the electronic structure, band gap, Fermi level (chemical potential), and electronic conductivity can be controlled with great precision. Silicon doped or alloyed with boron (BSi) is particularly interesting. Boron improves electrical conductivity in silicon, which improves rate capability for cycling. In single nanometer-scale BSi, interfacial dipoles and dative bonding change the electrostatic landscape and enable molecular control at the nanoparticle surface.^30^ While early reports on micrometer-sized BSi conclude that boron incorporation has little effect on silicon cycle stability, later reports on micrometer-sized, hundreds of nm sized, and thin-film BSi electrodes indicate that indeed boron has a sizable effect on improving electrochemical cycling.^31−33^ These promising reports combined with the wealth of controllable parameters relevant to battery operation offer a promising frontier to enable high silicon content in LIBs with a potential for rapid scale-up.

Here, we adopt this strategy and utilize novel single-nanometer-scale BSi alloy nanoparticles (NPs) as the anode active material for LIBs. We show that the energy of the Si 2*p* orbital (a proxy for the chemical potential) shifts to lower energies when B is incorporated into the Si NPs. We leverage the highly Lewis-acidic interface unique to our single nm BSi NPs to create a chemically modified, Li-ion containing surface that enables simple processing and a form of prelithiation. These two innovations extend the lifetime of >70 wt % silicon anodes from 350 cycles in pure silicon to >1000 cycles when paired against an NMC811 cathode in a traditional carbonate electrolyte. Remarkably, these anodes have almost no impedance gain through the 1000 cycle experiment and have little morphological change or SEI growth as well. This strategy marks a considerable improvement in stabilizing the Li_*x*_Si interface against parasitic chemistries in LIBs.

BSi NPs are synthesized using plasma-enhanced chemical vapor deposition (PECVD) (see Experimental Section in the Supporting Information).^34^ By leveraging the kinetic growth process of PECVD synthesis, we can produce crystalline BSi NPs with free carrier densities >10^20^ cm^–3^, well beyond the limits of traditional boron doping (∼10^17^ cm^–3^).^34−36^ From inductively coupled plasma optical emission spectroscopy (ICP-OES) measurements, our BSi particle composition is B_1_/Si_0.97±0.1_ or ∼50 at% boron. Scanning tunneling electron microscopy (STEM) images displayed in Figure 1a show that our BSi NPs have a single size distribution, with an average diameter of *d* = 7.2 nm. In close agreement, Scherrer broadening analysis results from X-ray diffraction (XRD) measurements indicate an average particle diameter of *d* = 6.5 nm (Figure S1). We will refer to these particles as 6.5 nm diameter particles, as Scherrer broadening captures the NP ensemble where STEM does not. The small NP diameter is chosen both to increase colloidal stability in the composite slurry and to prevent NP fracture during electrochemical cycling.^37^ The electron energy loss spectroscopy (EELS) data displayed in Figure 1b confirm the presence of both B and Si, and positional mapping from STEM-EELS in Figure 1c,d shows a mostly uniform distribution of B in Si, though some phase segregation can be seen in Figure 1c.

**Figure 1:**
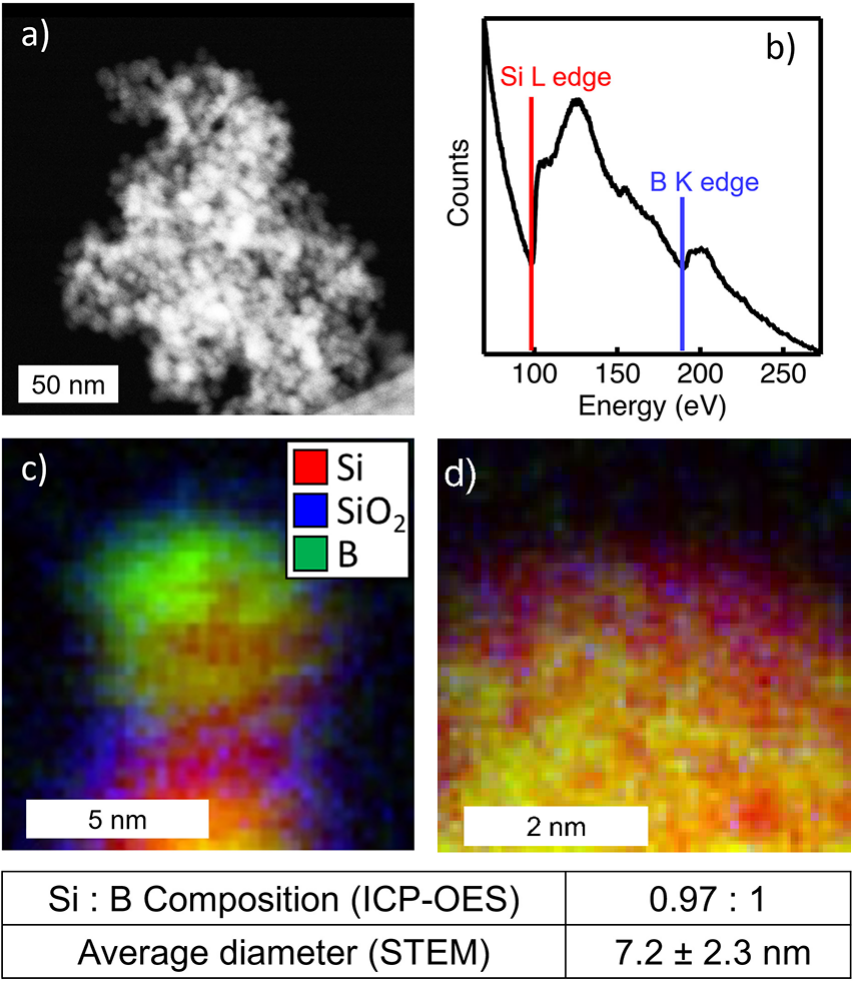
(a) STEM image of BSi NPs. (b) EELS of BSi. (c) STEM-EELS map of a BSi NP. Green indicates boron, red indicates Si, and blue indicates SiO_2_. (d) STEM-EELS map with a higher magnification. The table below lists the values of the composition and the average diameter.

For structural, chemical, and electronic characterization, we perform a combination of diffuse reflectance infrared Fourier transform spectroscopy (DRIFTS), XRD, and X-ray photoelectron spectroscopy (XPS). These data are summarized in Figure 2. The XPS data of B 1*s* for as-synthesized BSi NP powder displayed in Figure 2b confirm the presence of boron for the BSi where none is seen in the pure Si NP powder sample. Where the B *1s* peak center for elemental boron is 189.4 eV, the B 1*s* peak here is centered at 188.2 eV. This shift is consistent with a boride-like electronic environment, where electron density is donated from silicon to boron. The Si 2*p* core level spectra in Figure 2d show the presence of the expected Si^0^ species as well as a peak 0.95 eV higher in energy than Si^0^ for both pure Si and BSi. This envelope contains both Si^1+^ and Si–B, making deconvolution of the two species very difficult.^38,39^ Qualitatively, however, Si^1+^/Si–B is much more prominent in BSi than pure Si as we would expect for a 50% BSi alloy compared to pure Si NP with minor surface oxidation. From the DRIFTS spectra plotted in Figure 2c, the presence of both surface boron and surface silicon is evident from the surface hydride vibrational modes at 2550 cm^–1^ (*B–H) and 2080 to 2150 cm^–1^ (*Si–H_*x*_, *x* = 1, 2, or 3), respectively. From XRD (Figure S2), a 0.5% contraction of the diamond cubic structure—as measured by an increase in 2θ of the (111) diffraction peak, compared to pure Si NPs—confirms the incorporation of boron into the crystalline silicon lattice as well as the surface. These compositional, structural, and chemical characterization methods provide strong evidence that the material is a crystalline BSi alloy NP with surface *B–H_*x*_ and *Si–H_*x*_ sites.

**Figure 2:**
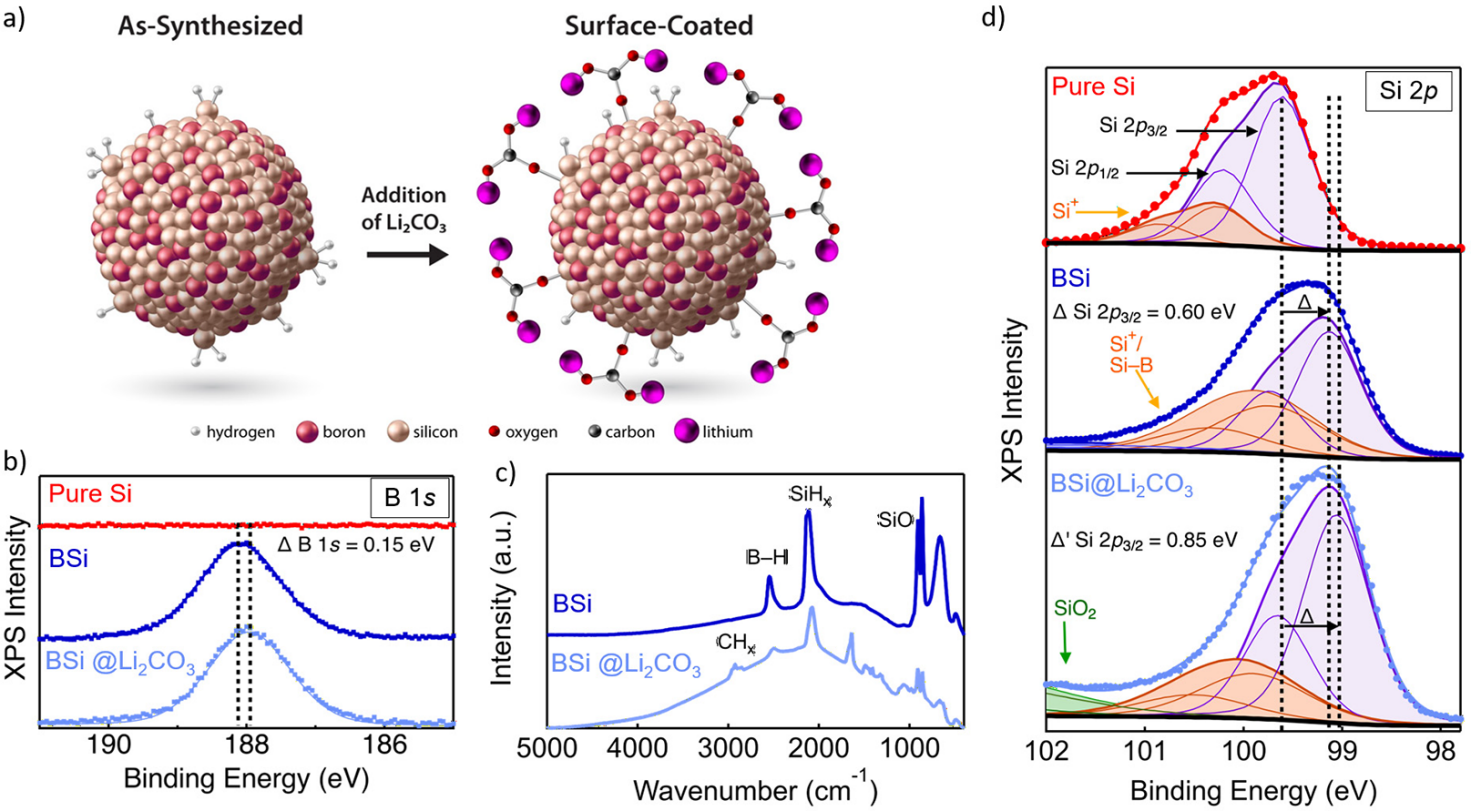
(a) Schematic illustration showing surface characteristics of as-synthesized, hydride-terminated BSi NPs powder and BSi@Li_2_CO_3_ NPs. (b) X-ray photoelectron spectroscopy of the B 1s core level for pure silicon NP powder (red), BSi NP powder (dark blue), and BSi@LiCO_3_ NP powder (light blue). (c) DRIFTS spectra of both as-synthesized BSi NP powder (dark blue) and surface-coated BSi NP powder, BSi@LiCO_3_ (light blue). (d) X-ray photoelectron spectroscopy of the Si 2p core level for pure silicon NP powder (red), BSi NP powder (dark blue), and BSi@LiCO_3_ NP powder (light blue).

Boron’s valence shell in the neutral state contains three electrons and silicon’s contains four. To achieve charge neutrality in the BSi lattice, boron acts as a shallow electron acceptor that liberates delocalized positive charges (holes) into the valence band. These holes move the Fermi level near the valence band edge of the semiconductor. This property is the operational premise for creating internal electric fields in silicon-based *pn* junctions. The presence of these positive charge carriers in our BSi NPs is evident from DRIFTS (Figure 2c). The DRIFTS spectrum shows a broad absorption feature between ∼400 and 2500 cm^–1^, which we have previously shown to be a localized surface plasmonic resonance (LSPR).^40,41^ LSPR arises from the collective oscillation of delocalized charge carriers in the Si valence band (the magnitude and peak position are proportional to the doping density).^42^ From XPS, the presence of B and its associated charge carriers in the BSi NPs shifts the Si 2*p*_3/2_ peak to a lower energy by ΔSi 2*p*_3/2_ = 0.60 eV from its position in pure Si. Moreover, where the B 1*s* peak center for elemental boron is 189.4 eV, the B 1*s* peak here is centered at 188.2 eV. This shift is consistent with a boride-like electronic environment where electron density is donated from silicon to boron which is expected when boron behaves as shallow acceptor.^43^ Overall, these data show a clear divergence in the electronic structure from pure silicon, which is consistent with the incorporation of an aliovalent species into the lattice.

Our previous reports on pure silicon nanoparticles demonstrate that these PECVD materials are highly reactive and that is essential to reduce the reactivity through molecular surface functionalization before they can be used as the active material in a LIB anode.^17,22,44−47^ The three-coordinate surface boron sites are strongly Lewis acidic and will interact with molecular species that have electron-donating groups via dative interactions.^30^ As a form of SEI engineering and prelithiation, we functionalized the surface of BSi NPs with Li_2_CO_3_ (BSi@Li_2_CO_3_) by simply mixing BSi NPs with a Li_2_CO_3_/NMP solution (Figure 2a). Here, the Li_2_CO_3_ mass is ∼10% of the BSi NP mass in the composite slurry. We choose Li_2_CO_3_ because it is a common inorganic SEI component,^48,49^ CO_3_^2–^ forms a strong dative bond with surface boron, and Li_2_CO_3_ provides an additional source of Li^+^. We note that if the BSi surface is not functionalized, these NPs will form a low-density gelatinous solid when mixed with a polymeric binder solution. The DRIFTS spectrum of BSi@Li_2_CO_3_ presented in Figure 2c reveals the same *Si–H_*x*_ and *B–H_*x*_ features as the BSi, but additional Li_2_CO_3_ (1470 cm^–1^) and NMP (2900 and 1750 cm^–1^) appear along with evidence of minor silicon oxidation (1100 and 2250 cm^–1^). In addition, the LSPR absorption onset is shifted to higher energy (4000 cm^–1^), which, according to the modified Drude equation,^42,50^ indicates an increase in the free carrier density. XPS measurements show an additional low energy shift of the Si 2*p*_3/2_ peak by 0.25 eV from the BSi making a total peak shift from pure Si (Δ′ Si 2*p*_3/2_) 0.85 eV. The position of the B 1*s* peak for BSi@Li_2_CO_3_ also shifts to lower energy compared to BSi by ΔB 1*s* = 0.15 eV, consistent with silicon donating additional electron density to boron making the BSi NP more *p*-type in nature. The increase in positive carrier density from Li_2_CO_3_ functionalization indicates that CO_3_^2–^ tightly binds to the BSi surface, which lowers the acceptor energy level of surface boron enough to liberate additional free carriers. We have reported a similar surface-doping effect on B-doped Si NPs previously.^34^

BSi@Li_2_CO_3_ slurries are prepared from colloidal dispersions of the 6.5 nm BSi@Li_2_CO_3_ NPs in NMP. Carboxylic acid-terminated single-walled carbon nanotubes (SWCNTs–COOH) are added as a conductive additive. SWCNTs–COOH are chosen both for their colloidal stability in NMP—which helps to maintain homogeneity of the anode—and their high aspect ratio, which reduces electronic isolation in the composite anode.^51,52^ A polyimide binder (PI) is used to adhere the composite and preserve the mechanical integrity of the anode during cycling. The composition of the electrode is 78% BSi, 11% PI, 5.5% SWCNTs–COOH, and 5.5% Li_2_CO_3_. Overall, the sequential addition of four components to NMP at room temperature is a fast and easy slurry preparation process that offers a promising route for high-throughput production of BSi-based composite LIB anodes. As a reference point, we also prepared silicon anodes from pure silicon nanoparticles with an average NP diameter of 6 nm. The composition of these electrodes is 76 wt % Si, 12 wt % PI binder, and 12 wt % SWCNTs. The synthesis and characterization of these electrodes has been described previously.^46^

Figure 3a shows the data for half-cell cycling of a BSi@Li_2_CO_3_ anode with 1.2 M LiPF_6_ in 3:7 ethylcarbonate:ethylmethyl carbonate with 3 wt % fluoroethylene carbonate (GenF3) as the electrolyte and Li metal as the counter electrode. The composite anode produces a first cycle delithiation gravimetric capacity of 1250 mAh/g from lithiating to 0.01 V and delithiating to 1.5 V and a Coulombic efficiency (CE) of 74%. The second and third cycles deliver nearly the same capacity with CE’s of 98.7% and 99.2%, respectively. The fast convergence of the CE to near 100% indicates that the BSi is quickly and effectively passivated, electrolyte wetting is fast and complete, and the anode does not lose active material in these early cycles. While the measured specific capacity is lower than expected for a pure silicon electrode of the same composition (2800 mAh/g), boron does not alloy with Li; thus, we expect that the BSi theoretical capacity to be lower than that of pure Si.^33^ When accounting for only the BSi mass in the electrode, the BSi NP alloy delivers 1800 mAh/g_BSi_ (Figure S3) corresponding to a maximum stoichiometry of Li:BSi around 2:1. Since silicon accounts for half of the BSi NP composition, the Si to Li stoichiometry in the fully lithiated state in BSi is nearly the same as pure silicon (∼4:1, Li:Si) which agrees well with analyses on BSi thin films.^33^ It is therefore likely that lithium is stored in BSi in the same way as pure Si: as a random alloy.

**Figure 3:**
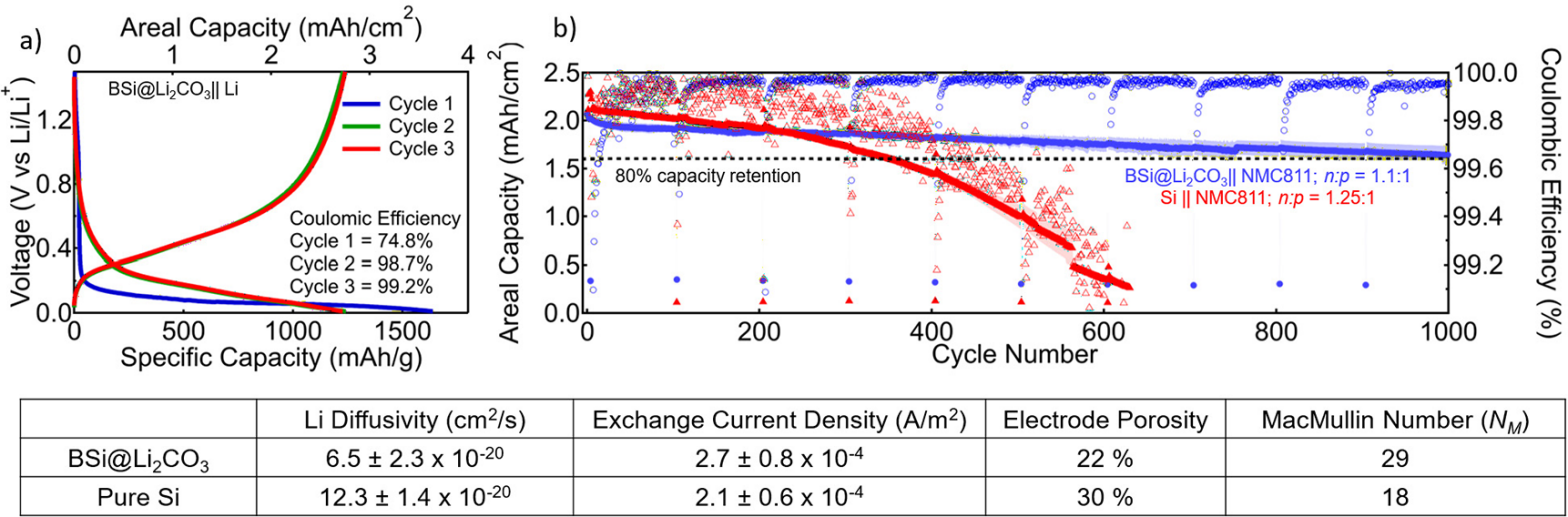
(a) Electrochemical cycling data for BSi@LiCO_3_ anodes in GenF3 electrolyte against Li metal. (b) Average electrochemical capacity measurements for two BSi@Li_2_CO_3_ || NMC811 batteries (blue, circles) and two Si || NMC811 batteries (red, triangles). The shaded region represents ±1σ from the mean. The Coulombic efficiencies for each data point are shown as the open circles of the same colors. The dashed red line is drawn at 80% of the initial capacity. The table below lists material and electrode-level properties determined from the galvanostatic intermittent titration technique and electrochemical impedance measurements.

The cycle performance of these electrodes in a full cell configuration against a capacity matched NMC811 cathode (BSi@Li_2_CO_3_||NMC811) is shown in Figure 3b. These anodes were harvested from half cells after three cycles, leaving the electrodes in the delithiated state. We do not consider these anodes prelithiated as the anode is completely delithiated to 1.5 V vs Li/Li^+^. Instead, these anodes are “preformed”, which reduces the irreversible first cycle losses but does not add active Li to the battery inventory. Two separate coin cells for each BSi@Li_2_CO_3_ || NMC811 and pure silicon anode batteries (Si || NMC811) were cycled 1000 times. The average areal capacity and CE as well as the variance (±1σ from the mean) from these tests are shown in Figure 3b. The anode capacity is defined by the reversible capacity on the third formation cycle. The cathode capacity is defined by the Cell, Analysis, Modeling, and Prototyping facility at the Argonne National Laboratory. The *n*:*p* ratio refers to the ratio between the anode capacity (*n*) and cathode capacity (*p*).

From the electrochemical cycling data in Figure 3b, the initial areal capacity of the BSi@Li_2_CO_3_ || NMC811 cell is 2.08 mA/cm^2^ with an anode specific gravimetric capacity of 1210 mAh/g (Supporting Information, Figure S4) amounting to a total cell stack energy density of 260 Wh/kg. After two additional cycles, the average CE rises to <98% and <99.9% after 40 cycles. Similarly, Si || NMC811 displays CE values above 99.8% and an areal capacity of 2.2 mAh/cm^2^. The variance in areal capacity for both electrode sets never exceeds more than 5% of the average capacity, indicating reasonable cell-to-cell reproducibility. Rate capability tests of BSi@Li_2_CO_3_ || NMC811 (Supporting Information, Figure S3) show that the battery delivers 70% of its available capacity at a current density of 1.22 mA/cm^2^ (1 C) and 40% of its’ capacity at 4.9 mA/cm^2^ (4 C). Similar rate capabilities were observed for Si || NMC811.^46^ After completing 1000 cycles, the BSi@Li_2_CO_3_ || NMC811 retains slightly more than 80% of its initial capacity, where the Si || NMC811 reaches only 310 cycles before the 80% threshold. We note that these Si || NMC811 batteries can achieve 1000 cycles while retaining ∼80% of their capacity, but they require a large excess of Li from prelithiation.^46^ The remarkable performance and high CEs of the preformed BSi@Li_2_CO_3_∥NMC811 without prelithiation is consistent with the reduced surface reactivity of BSi@Li_2_CO_3_.

While capacity retention is critical for secondary batteries, power retention through impedance minimization is equally important. To this end, we track the area specific impedance (ASI) by performing a hybrid pulse power characterization (HPPC) measurement every 100 cycles. The changes in ASI (ΔASI) and absolute ASI data for BSi@Li_2_CO_3_ || NMC811 and Si || NMC811 are shown in Figure 4a. The ASI of BSi@Li_2_CO_3_ || NMC811 at cycle 0 is 123 Ω cm^2^, and the Si || NMC811 at cycle 0 is 42 Ω cm^2^. The difference in ASI between BSi@Li_2_CO_3_ || NMC811 and Si || NMC811 at cycle 0 could be related to the 5 times slower Li diffusivity compared to pure Si at 7.6 × 10^–20^ m^2^/s and 4.0 × 10^–19^ m^2^/s, respectively. (Note: These Li diffusivity values are lower than the typical measured values. Additional discussion on our GITT data is provided in the Supporting Information, Figures S5 and S6.) However, the exchange current densities for BSi and pure Si are very similar, 2.5 × 10^–4^ A/m^2^ and 1.2 × 10^–4^ A/m^2^ (see the Supporting Information, Figures S5–S7, and the associated discussion for more details), and the rate capability of BSi@Li_2_CO_3_ || NMC811 is commensurate with pure silicon electrodes (Supporting Information Figure S3).^46^ More likely, the elevated initial ASI in BSi@Li_2_CO_3_ || NMC811 is related to the more tortuous and compact BSi@Li_2_CO_3_ electrode microstructure (Figure S7 and associated discussion), which can be overcome with appropriate electrode architecture engineering. As the cycle test progresses, however, the ASI of Si || NMC811 increases rapidly, with a total gain of 77 Ω cm^2^ to a final value of 119 Ω cm^2^ through 600 cycles. In contrast, BSi@Li_2_CO_3_ || NMC811 shows no increase in ASI and even a slight decrease throughout the 1000 cycle experiment. These data indicate that cycling BSi@Li_2_CO_3_ does not impact ionic or electronic transport in the anode. Indeed, the BSi@Li_2_CO_3_ appears to be even more stable than PECVD silicon nanoparticles buried in carbon.^17^

**Figure 4:**
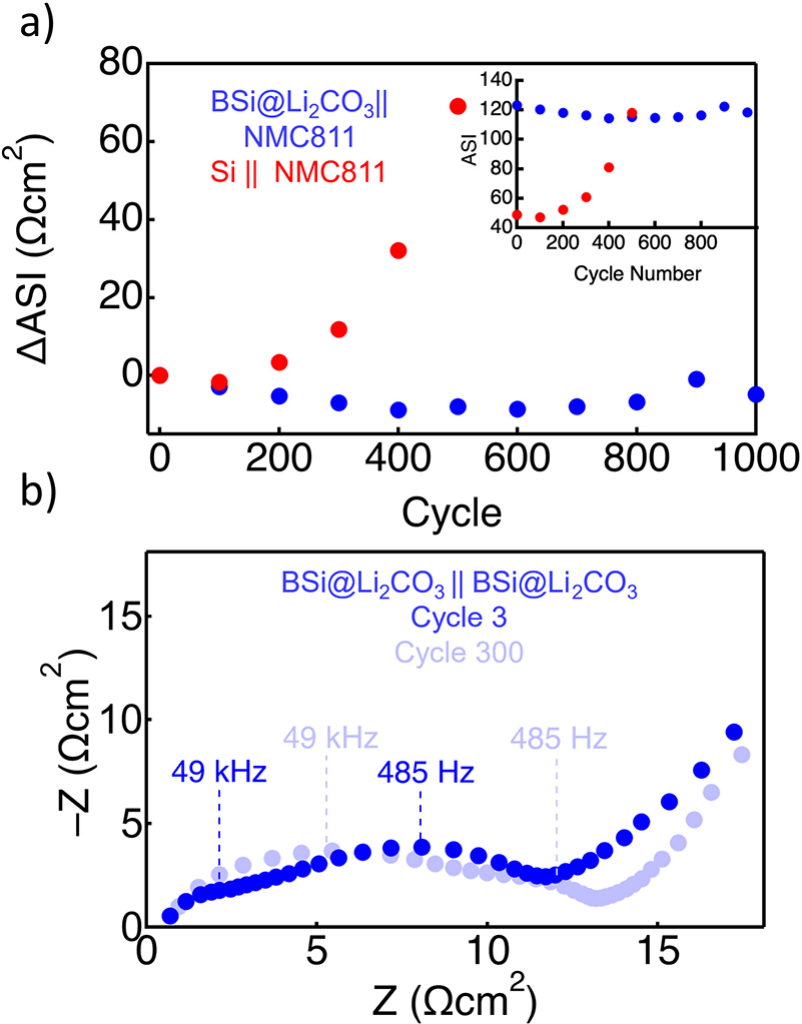
(a) Change in the area specific impedance (ΔASI) for BSi@Li_2_CO_3_∥ NMC811 (blue) and Si∥NMC811 (red) at a cell potential of 3.2 V. These data were derived from hybrid pulse power characterization steps every 100 cycles. The inset shows the absolute ASI values of the cell. (b) Nyquist plots of BSi@Li_2_CO_3_ symmetric cells after the formation cycle (blue) and after 300 cycles in a full cell configuration against NMC811 (light blue). Cycle 0 is the anode in a full cell after preformation in a half cell.

To verify this finding, we performed electrochemical impedance spectroscopy (EIS) on symmetric BSi@Li_2_CO_3_||BSi@Li_2_CO_3_ cells, which eliminates the possibility of impedance contribution from the cathode or Li counter electrode. These measurements were before and after 300 electrochemical cycles against NMC811 where “Cycle 0” is BSi@Li_2_CO_3_ electrodes that have been preformed in a half cell and not cycled in a full cell. The Nyquist plots for these cells are shown in Figure 4b. The data in Figure 4b display two electrochemical processes occurring in each set of anodes in different frequency regimes. Previous EIS studies have identified the fast process as the double layer charging and the slow process as charge transfer.^53^ Here, we label the frequency at the maximum −Z value (1/τ, τ = RC time constant) for the anodes to visualize changes in the electrode kinetics. From graphical analysis of the real axis of the Nyquist plot which represents cell resistance,^54^ the resistance increases for double layer charging after 300 cycles, but it decreases for charge transfer such that the overall anode resistance (the sum of the diameters of the semicircles on the *x*-axis for both processes) is nearly unchanged from 12 Ω cm^2^ to 14 Ω cm^2^ after 300 cycles. The change in resistance for double layer charging or charge transfer from 3 to 300 cycles is >5 Ω cm^2^ indicating that while the individual processes are slightly different after cycling, the change is not significant. Moreover, the frequency at −Z maximum is nearly identical for each process before and after cycling indicating that the kinetics of charge transport and ion transport are virtually unchanged for each process as well. These impedance data unambiguously show that electrochemical cycling minimally impacts the transport properties of the BSi@Li_2_CO_3_ anode, suggesting that electrolyte degradation is virtually nonexistent.

To corroborate these electroanalytical data, we track the morphological evolution of BSi@Li_2_CO_3_ anodes through scanning electron microscopy (SEM) and energy dispersive X-ray spectroscopy (EDS) measurements before and after 1000 cycles (Figure 5 and Supporting Information Figures S8–S10). SEM images of the pristine anode reveal a densely packed monolithic structure with an average thickness of ∼25 μm (a detailed discussion of the microstructure can be found in the Supporting Information). The cracks in the electrode are the result of solvent evaporation after electrode printing. Astoundingly, after 1000 lithiation/delithiation cycles, the anode microstructure remains almost entirely unchanged. The thickness of the cycled electrode is nearly equal to the pristine electrode, and very little deformation in the coating is evident, in contrast to our pure silicon electrodes.^46^ EDS maps of the corresponding SEM images (Figures S8 and S9) show a slight increase in the carbon and oxygen intensities (as well as the appearance of fluorine and phosphorus) compared to the as-prepared electrode, but the elemental distribution is largely unchanged. In addition, no apparent distortion or delamination of the active mass is visible after 1000 cycles (Supporting Information, Figure S11). Indeed, following the formation cycles, these BSi@Li_2_CO_3_ electrodes appear to form little, if any, additional SEI during extended cycling.

**Figure 5:**
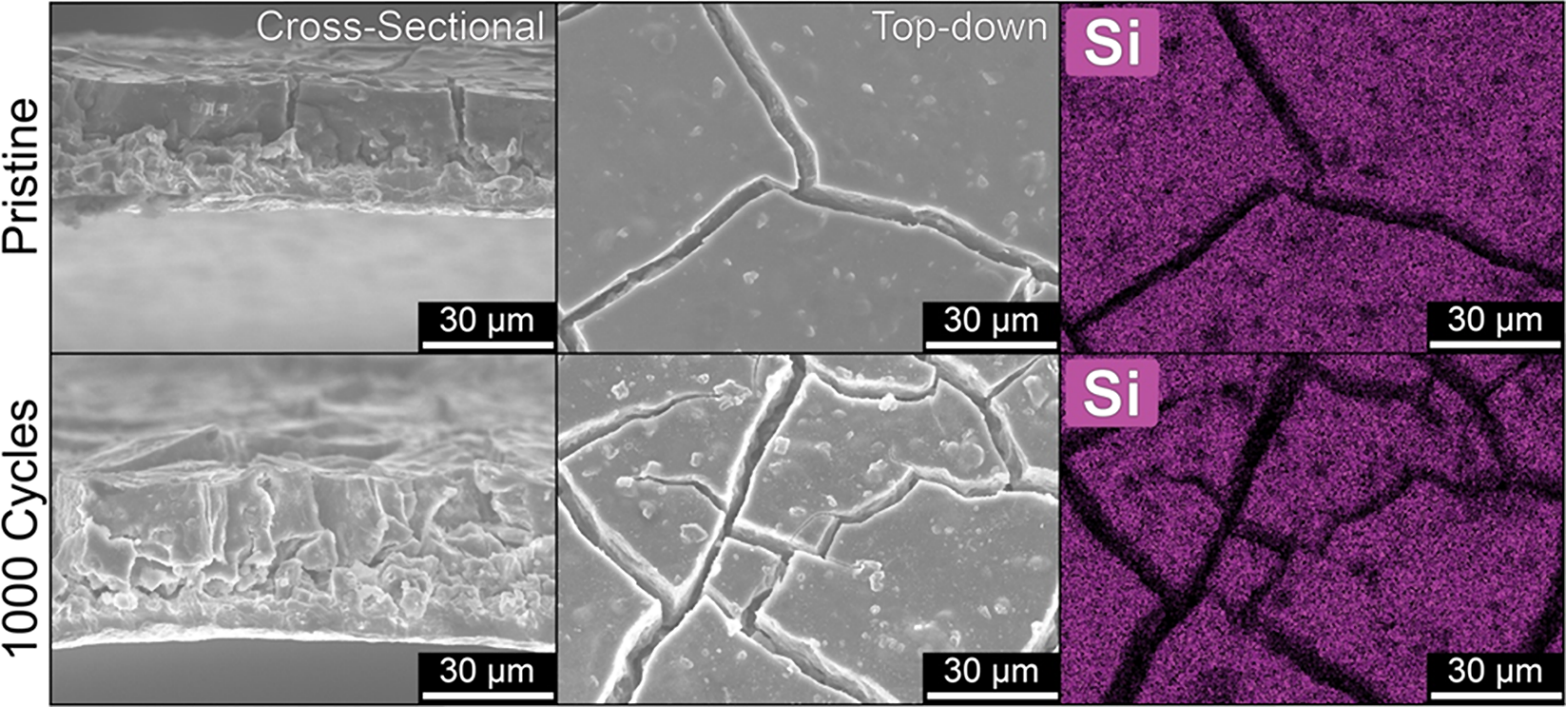
Cross-sectional (left) and top-down (middle) SEM images of BSi@Li_2_CO_3_ composite anodes are shown for both pristine (top) and cycled (bottom) anodes. EDS mapping data showing Si distribution (right) correspond to top-down SEM images.

Boron/silicon alloy nanoparticles are a new frontier of active materials for LIB applications. The unique chemical and electronic structures of these particles enable new parameters to tune for improving chemical stability against electrolyte decomposition, improving electrical conductivity, and capturing the highest energy density for silicon-based active materials. While we have demonstrated that these powders are easy to process, have high Coulombic efficiencies at early cycles, can be prelithiated by simply adding a Li_2_CO_3_ salt, and form little SEI, there are salient questions that remain. For example, the specific origin of the improved cycling stability is unclear. It is possible that enriching the BSi surface with Li_2_CO_3_ passivates the lithiated BSi against parasitic chemistry, but the free electrons from *p*-type doping may also play a role. Moreover, the structure of the SEI itself may be entirely different than the SEI of pure silicon, as Li_2_CO_3_ is tightly bound to the surface of the BSi and is only one of many components of the SEI of pure Si. Such an alteration may also impact the electrostatic energy profile within the SEI which could more effectively screen the highly reducing potential of the LiBSi surface. In addition, the structural and compositional evolution of this alloy may change with cycling. Silicon undergoes amorphization upon lithiation and delithiation. Since these particles are metastable (above the thermodynamic solubility limit), boron migration is likely to occur with lithiation/delithiation. Indeed, the diffusivity of B in Si is on the order 10^–13^ cm^2^/s at equivalent electrochemical energy values of ∼0.1 V from the equilibrium potential.^55^ If indeed boron does redistribute throughout the BSi NP structure, then atomic distribution may also be the origin of the enhanced stability. Finally, the atomic ratio of B to Si is not optimized. It is possible that significantly less boron is needed to impart the same stabilizing effects as the 50 atom % BSi alloy used here. These and other questions will be the topic of our follow-up studies on this promising new class of materials for negative alloy anodes in LIBs.

In summary, we have adopted an uncommon strategy to passivate the surface of Li_*x*_Si by alloying Si with an aliovalent element—boron. This alloy displays considerably different physical properties than pure silicon, namely, a reduced chemical potential through *p*-type doping and a highly Lewis acidic surface. These particles are fabricated into composite electrodes by simply mixing four components into a slurry at room temperature and printing onto a current collector and therefore could readily be adopted by using standard manufacturing practices for high-volume production. These majority silicon electrodes display remarkable cycle stability by reaching 1000 cycles with >80% capacity retention and exhibit little or no impedance gain or SEI growth. Changing the content and identity of aliovalent dopant atoms in silicon offers a new form of control over silicon active materials for lithium-ion battery technology.

## Supporting Information Available

Experimental details; XRD and additional electrochemical analysis; Li solid-state diffusion discussion; electrode morphology analysis; SEM, EDS, and electrode photographs (PDF)
