# Supplementary material for: Boron–Silicon Alloy Nanoparticles as a Promising New Material in Lithium-Ion Battery Anodes
Source: ACS Energy Lett. 2024 May 2;9(6):2492–9. doi: 10.1021/acsenergylett.4c00856 (PMC11190990; doi:10.1021/acsenergylett.4c00856)
Supplement: Supplementary file 1 — nz4c00856_si_001.pdf [file nz4c00856_si_001.pdf]

Supporting Information for:

Boron-Silicon Alloy Nanoparticles as a Promising New Material in Lithium-Ion Battery Anodes

Gregory F. Pach[1,*], Pashupati R. Adhikari [1], Joseph Quinn[2] , Chongmin Wang[2] , Avtar Singh[3] , Ankit Verma[3], Andrew Colclasure[3], Jae Ho Kim[1,4] , Glenn Teeter[5] , Gabriel M. Veith[6], Nathan R. Neale[1,7] , and Gerard M. Carroll[1, *]

1. *Chemistry and Nanoscience Center, National Renewable Energy Laboratory, 15013 Denver West Parkway, Golden, Colorado 80401, United States*
2. *Environmental Molecular Sciences Laboratory, Pacific Northwest National Laboratory, Richland, Washington 99342, United States*
3. *Energy Conversion and Storage Systems Center, National Renewable Energy Laboratory, Golden, Colorado 80401, United States*
4. *Department of Nanoenergy Engineering, Pusan National University, Busan 46241, Republic of Korea*
5. *Materials Sciences Center, National Renewable Energy Laboratory, 15013 Denver West Parkway, Golden, Colorado 80401, United States*
6. *Chemical Sciences Division, Oak Ridge National Laboratory, Oak Ridge, Tennessee 37831*
7. *Renewable and Sustainable Energy Institute, University of Colorado Boulder, Boulder, Colorado 80309, United States*

Experimental Section

BSi@Li_2CO_3 Synthesis and Electrode Fabrication.

Radio Frequency Non-Thermal Plasma Synthesis of Hydride-Terminated BSi NPs:
6.5 nm BSi nanoparticles were produced by a nonthermal plasma method using a capacitively coupled 13.56 MHz RF plasma. Process gases SiH_4 (9 sccm) and B_2H_6 (14 sccm) accompanied by carrier gases of Ar (20 sccm), H_2 (70 sccm), and He (81 sccm) were flowed through a 25 mm OD/19 mm ID quartz reactor tube. 250 W was supplied to the electrode using an Advanced Energy Cesar 136 generator through an Advanced Energy VM1000 matching network. Throughout the reaction the pressure was maintained at 3 torr. BSi nanoparticles were collected downstream from the plasma on a 400-mesh stainless steel filter and transferred air-free to an inert-atmosphere glovebox for subsequent processing. CAUTION! Air oxidation of BSi nanoparticles generates toxic B_2H_6 gas. Pure silicon NPs were synthesized through PECVD process as well. The details are described elsewhere.[1]

Slurry Fabrication:
BSi@Li_2CO_3: 40 mg SWCNTs-COOH (purchased from Cheap Tubes) were dispersed in 3 mL NMP and sonicated for 30 minutes. 20 mg lithium carbonate was then added to the solution and

vortexed. Next, 290 mg BSi NPs was added, and the solution was stirred on a hotplate at room temperature for ~1 hr. Finally, 20 mg of Ensinger polyimide P84 binder was added and the slurry was left stirring at room temperature overnight. The slurry was then sealed under Ar and brought outside the glovebox and mixed using a dual axis Kurabo KK-250s planetary mixer.

Pure silicon: Pure silicon slurry preparation has been described elsewhere.[1] Briefly, we used silicon nanoparticles with diethylene oxide (PEO) covalently bound to the surface as the silicon source material. The PEO provides colloidal stability to the Si which enables a homogeneous slurry. The silicon nanoparticles are suspended in NMP followed by the addition of SWCNT and then a PI/NMP solution. The slurry was mixed on a stir plate for 16 hours in an Ar atmosphere.

Anode fabrication:

Anode fabrication was performed in an air-free, Ar-filled glovebox. After mixing, the slurry was blade cast onto a copper foil current collector using a step height of 250 μm at a speed of 1 cm/s. After casting the slurry, the electrode was dried at 150 °C for 4 hr under vacuum (10^{-2} torr). After drying, the anode was removed and annealed at 450 °C for 4 hr in a quartz tube furnace under flowing N_2. For the pure silicon electrodes, the PEO volatilizes above 350°C which leaves a silicon surface with a native oxide layer.[1]

Coin cell fabrication:

NP composite anodes were tested using 2032 coin cells. Composite anodes were initially formed in a half cell arrangement against Li metal foil, and subsequently disassembled and reassembled into full configuration against NMC811. BSi NP anodes were used as the negative electrode while 14 mm diameter circular punches of NMC811 were used as the positive electrode. Coin cells were assembled by attaching each electrode to a 16 mm diameter, 1 mm thick stainless-steel spacer with a stainless steel wave spring positioned behind it. The electrodes are separated by a 19 mm diameter circular punch of porous polypropylene separator (Celgard 2325). Each coin cell is injected with 2 aliquots of 20 μL GenF electrolyte (1.08 $LiPF_6$ in fluoro-ethylene-carbonate/ethylene-carbonate/ethyl-methyl-carbonate 1:2.7:6.3 by weight) onto either side of the separator before cells were sealed with a hydraulic crimper.

Characterization.

Electrochemical cycling:

Coin cells were tested in a Maccor model series 4000. After fabrication, coin cells rested at open circuit for 4 hours before electrochemical cycling. For half cells, coin cells are galvanostatically cycled at a rate of C/20 for 3 cycles between 0.01 and 1.5 V vs. Li/Li^+. For full cells, coin cells are first cycled in a half cell configuration and then reassembled into a full cell against a standardized cathode. The cathode used in these studies was provided by the Cell Analysis, Modeling, and Prototyping (CAMP) facility at Argonne National Laboratories. The cathodes consist of NMC811 (90%), Timcal C45 conductive carbon (5%), and Solvay 5130 binder (5%). The areal capacity of these cathodes is 2.5 mAh/cm^2 when electrochemically cycled between 3 – 4.2 V vs Li. Once assembled, the full cells are galvanostatically cycled at a rate of C/20 for 3 cycles between 3.0 and 4.2 V vs. Li/Li^+ and then cycled at C/3 for 1000 cycles. A HPPC cycle is performed every 100 cycles to measure the cell impedance.

The total mass considered for the cell stack energy density in Figure 3 (b) of the main text is described below.

$$Cell\ stack\ energy\ density = \frac{Energy\ (Wh)}{Anode\ mass_{Cu\ +\ composite} + Cathode\ mass_{Al\ +\ composite} + separator\ mass + Electrolyte\ mass}$$

Eq. 1

DRIFTS:
DRIFTS measurements were performed in an Ar-filled glovebox on a Bruker Alpha FTIR spectrometer. Measurements were taken on NP powders using gold-coated polished Si wafer substrates. Spectra were baseline-corrected using the concave rubber band correction method.

XRD:
XRD spectra were taken on a Rigaku Dmax diffractometer using Cu Kα radiation (λ = 1.54 Å). Toluene slurries of BSi NPs were deposited on Si zero diffraction substrates. NP diameters were calculated using Scherrer analysis ($D_{NP} = \frac{k \cdot \lambda}{w \cdot \cos(c)}$) with the shaping factor k = 1.1, X-ray wavelength λ = 1.54 Å, and w and c being the width and center of the Si (111) diffraction peak, respectively.

Electrochemical Impedance Spectroscopy.
EIS measurements were performed in a symmetric cell configuration (BSi@Li_2CO_3 || BSi@Li_2CO_3). A symmetric cell configuration removes possible EIS artifacts induced by the counter/reference electrode from this two-electrode configuration. These electrodes were completely delithiated prior to EIS measurements. For EIS measurements performed from "cycle 3", these electrodes were first pre-formed in half cells according to the procedure described above. The electrolyte used in these experiments is GenF.

SEM/EDS:
All SEM images were collected using a Hitachi 4800 microscope with a 15 kV accelerating voltage and a working distance of approximately 10-12 mm. Samples for cross-sectional SEM images were prepared by tearing an electrode (either before or after electrochemical cycling). The top-down images of the electrodes were collected with no further sample preparation steps. The elemental composition was determined at an accelerating voltage of 20 keV.

ICP-OES:
Inductively coupled plasma – optical emission spectroscopy (ICP–OES) (Thermo Scientific iCAP 7400 ICP-OES Duo) was performed to quantitatively characterize the Si : B ratio. Briefly, samples were weighed out in an argon filled glove box to avoid spontaneous ignition in air. Samples were removed from the glove box and immediately quenched by adding 18 MΩ DI water followed by 2 mL of nitric acid (Fisher Chemical, Trace metal grade) to oxidize the silicon nanoparticles. Approximately 2 mL of 5% hydrofluoric acid (diluted from concentrated HF solution – Sigma Aldrich, Trace metal) were added and allowed to complete the dissolution of the BSi sample.

Unreacted HF was observed to skew the silicon signal from etching the glass nebulizer of the HF. The error was estimated to increase the silicon content by 20 ppm which was accounted for in the data analysis.

XPS:
XPS measurements were performed in a Physical Electronics Phi VersaProbe III instrument using monochromatic Al-ka X-rays (hν = 1486.7 eV). High-resolution spectra were acquired with a pass energy of 55 eV. Curve-fitting of XPS spectra was performed in Igor Pro using a previously described approach where phase identification is facilitated by applying appropriate constraints on core-level binding energies and peak areas.[2] All XPS measurements were performed on NP powders.

STEM/EELS:
Scanning transmission electron microscopy (STEM) and electron energy loss spectroscopy (EELS) was performed on a monochromated FEI Titan TEM with a probe aberration corrector and a Gatan Image Filter (Quantum Spectrometer). For the STEM images the electron dose rate was ~$4.5x10^7$-$2x10^8 e \cdot Å^{-2} \cdot s^{-1}$. For EELS measurements, the Si $L_{2,3}$-edge and the B K-edge was measured with a dispersion of 0.1eV/ch, an energy resolution of ~1 eV, a pixel size of 0.3 nm, and an exposure time of 10 ms, and a collection semi angle of 44 mrad. The Si, SiO_2, B EELS maps were calculated from a model using Gatan Digital Micrograph software. The model used a power law background and was corrected for plural scattering. The cross-section model used Hartree-Slater for boron and standard reference spectrums for Si and SiO_2.

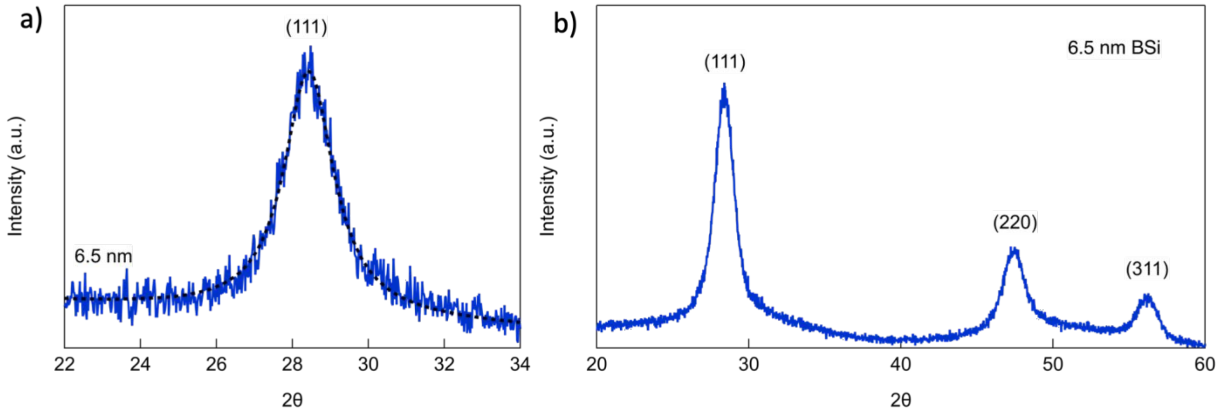

Figure S1. **a)** XRD spectrum of the (111) diffraction peak from PECVD-grown 6.5 nm BSi NPs. The dashed line shows the fit of the peak used for Scherrer analysis to subsequently determine the NP diameter. **b)** Extended XRD spectrum of the same BSi NPs in a).

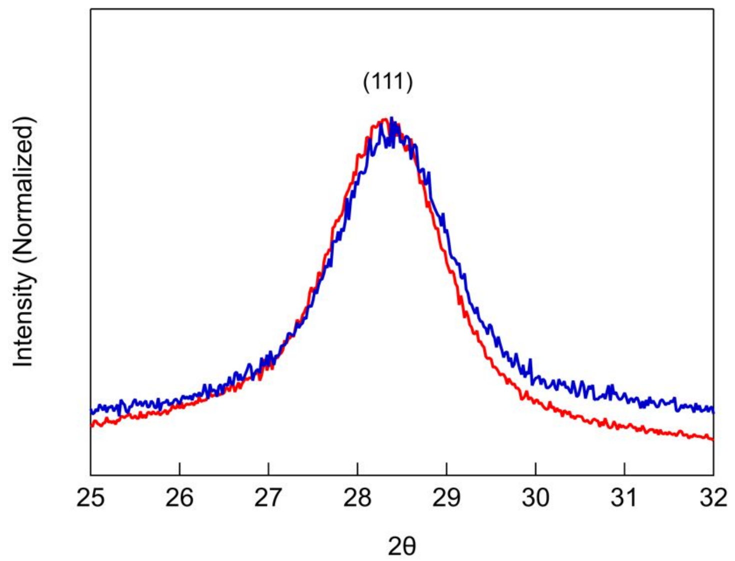

Figure S2: XRD spectrum showing the (111) diffraction peak for 6.0 nm PECVD-grown pure Si NPs (red) and 6.5 nm BSi NPs (blue).

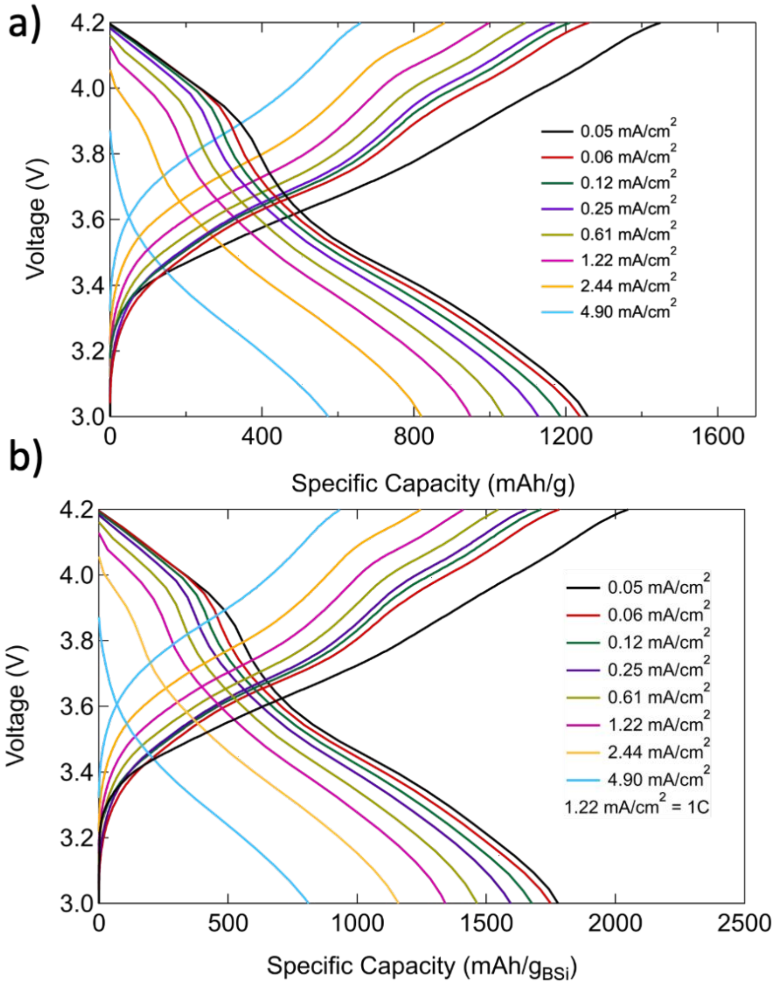

Figure S3: Rate capability data for BSi@Li_2CO_3 || NMC 811. These data are presented for the specific capacity of the anode **(a)** and for only the mass of the BSi **(b)**.

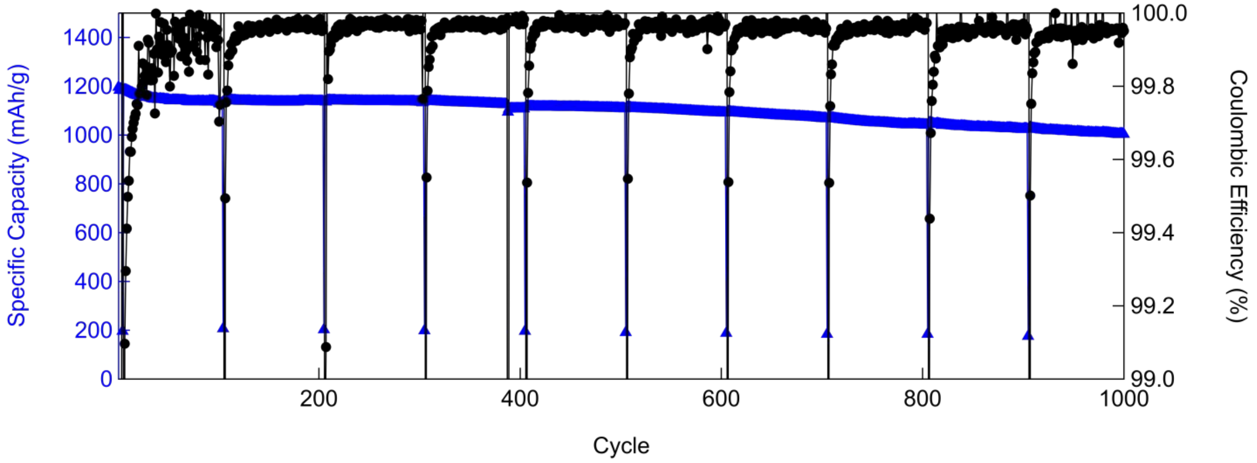

Figure S4: Specific capacity information of the BSi anode in the BSi || NMC811 in the cycled data presented in Figure 3a. Note that this data is from a single coin cell.

Li diffusivity and exchange current density.

To benchmark the Li diffusion and charge transfer kinetics in BSi@Li_2CO_3, we measure the Li diffusivity and exchange current density and compare them to pure Si. Galvanostatic intermittent titration test (GITT) is a standard electrochemical protocol to evaluate the kinetic (exchange current density) and transport properties (solid phase diffusivity) of electrode materials.[3] The protocol consists of short duration constant current pulse of low magnitude followed by a sufficiently long rest for electrode equilibration applied in a repetitive fashion between the cell operating voltage window. The advantage of this protocol lies in unraveling the state of charge (SOC) dependence of these properties as the electrode is lithiated/delithiated.

We apply the GITT protocol to our Si anodes in the half cell configuration after formation. Formation cycles consist of 3 C/20 cycles between 1.5V and 0.1V followed by a long rest prior to the start of GITT. The GITT protocol consists of 50 pulses each during Si lithiation and delithiation for a total of 100 pulses. A C/10 current is applied for 10 minutes followed by two hours of rest for relaxation. Figure S5a shows an exemplar staircase GITT voltage profile based on the stepped current-rest input on an undoped Si anode during discharge and charge. A zoomed in view on voltage and current evolution during a single rest-pulse-rest is shown in Figure S5b. Here, U^0 is the equilibrated open circuit potential at the end of the rest phase, $\eta_{kinetic}$ is the kinetic overpotential during the initial portion of the current pulse related to the exchange current density of the Li charge transfer process at Si anode|electrolyte interface and η_{conc} is the concentration overpotential during the latter portion of the current pulse related to solid phase diffusion of Li into the silicon host.

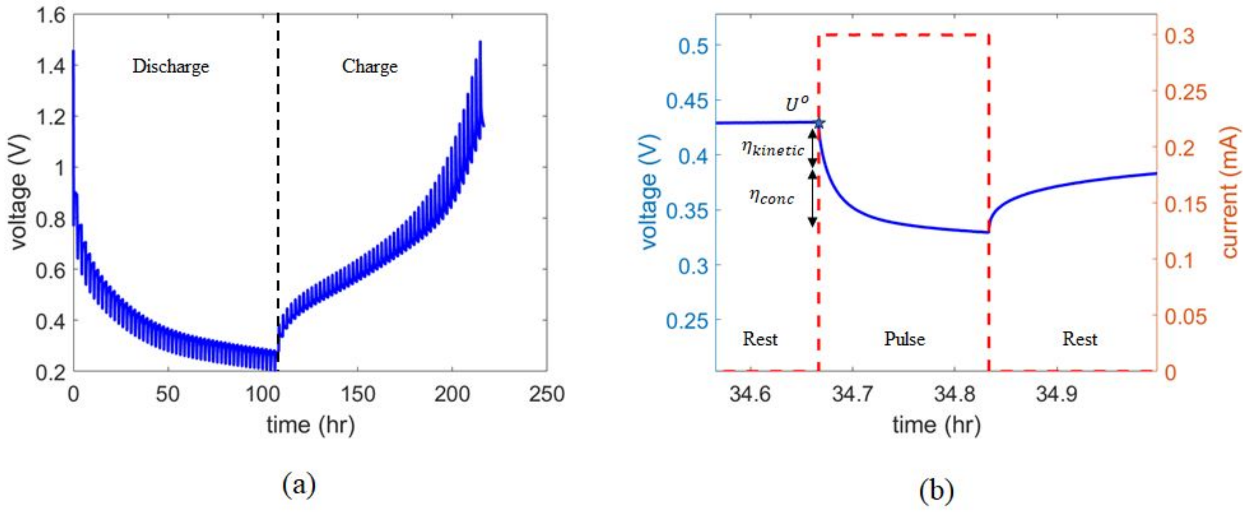

Figure S5: a) Full GITT voltage profile, and b) Magnified view of voltage and current profile in a single rest-current step.

Diffusivity, D_s, can be computed using the standard Weppner-Huggins equation:[4]

$$D_s = \frac{4}{\pi}\left[\frac{IV_m}{FS}\right]^2\left[\frac{dU^0(x)/dx}{dV(t)/d\sqrt{t}}\right]$$

Eq. 2

Here, I is the current pulse value, V_m is the molar volume of Si host, F is Faraday's constant, S is total electrochemically active area of the Si anode, $U^0(x)$ is the state of charge (x) dependent open circuit potential (OCP) of Si, V, t is the voltage and time during the current pulse. Molar volume is computed using molar mass and density of Si, $V_m = Molar\ Mass_{Si}/\rho_{Si}$. Active area can be computed using $S = 3\varepsilon_s L A_c/\mathrm{R}$ where ε_s is the Si volume fraction in the anode, L and A_c are electrode thickness and cross section area respectively, and R is the radius of Si nanoparticle taken to be 6.5 nm.

The remaining unknowns in diffusivity calculation are accurate derivatives of $U^0(x)$ and $V(t)$. We have a table of $U^0(x)$ from the end of rest equilibrated voltages after each current pulse. To calculate x, we need an initial estimate which is obtained by assigning the start of the GITT experiment to $x = 0$. This is reasonable because we are starting from an approximately fully delithiated state of silicon at potentials around 1.5V. Subsequent x values corresponding to each $U^0(x)$ value can be computed using $x_{i+1} = x_i + It_{pulse}/m_{Si}Q_{th}$, with $x_0 = 0$. Here, $t_{pulse}$is the 600s pulse duration, m_{Si} is the mass of active Si in the composite electrode and $Q_{th}$is the theoretical specific capacity of Si assuming full lithiation to $Li_{15}Si_4$. Once the tabulated x and U^0 values are obtained, numerical differentiation on piecewise polynomial fits is performed to obtain $dU^0(x)/dx$. V vs $\sqrt{t}$ during current pulse should show a linear correlation on excluding the abrupt potential decay/rise due to kinetic and ohmic overpotentials in the initial seconds of the pulse. An automated linear regression fit of V vs $\sqrt{t}$ is performed for each pulse by incrementally removing initial datapoints in the pulse until a high goodness of fit $R^2 > 0.99$ is obtained. Difference in the excluded voltage datapoints is then directly used to calculate the charge transfer kinetic overpotential, $\eta_{kinetic}$ for each pulse.
Exchange current density, i_o, is calculated using the linearization of Butler-Volmer in the small current density regime[3] and charge transfer kinetic overpotential, $\eta_{kinetic}$ obtained for each pulse using:

$$i_o S = \frac{\hat{\mathrm{R}}T}{F}\frac{I}{\eta_{kinetic}}$$

Eq. 3

Here, $\hat{\mathrm{R}}$ is the universal gas constant and T is the temperature of GITT test.

GITT measurements reveal that the average delithiation diffusivity of Li in BSi is 7.6 x 10^{-20} m^2/s, about one fifth that of our pure Si (4.0 x 10^{-19} m^2/s). The slower diffusion in BSi may be related to the lattice contraction observed upon alloying with boron which inhibits Li transport. However, these diffusivities are relatively similar (Li diffusivities range more than ten orders of magnitude) which suggests that the mechanism of Li diffusion in BSi is the same as pure Si. Interestingly, the exchange current density – an interface property that measures height of the activation barrier for reversible electron transfer at equilibrium – is nearly identical for BSi and pure Si at 1.2 x 10^{-4} A/m^2 and 2.5 x 10^{-4} A/m^2, respectively. So, despite the BSi interface being densely packed with boron and Li_2CO_3, the relevant electron transfer kinetics are unaffected. Overall, boron alloying in silicon appears to have little impact on the diffusion and charge transfer kinetics.

It is imperative to highlight some of the challenges of our GITT analysis and the rationale for low diffusivity and exchange current density magnitudes of our nanosized Si anodes. Typical Si diffusivities can span a wide order of magnitude with major clustering in the 10^{-16} – 10^{-18} m^2/s range.[5, 6] Our GITT analysis relies on no consideration of volume and structural changes in the electrode material which can happen with large expansion of Si. Furthermore, our composite anodes are composed of nm sized Si particles which results in high theoretical active area $S \propto 1/R_{Si}$. Coupled with low estimated porosity of the Si anodes (22-30%), possible agglomeration of Si nanoparticles to form larger particles and pore filling with solid electrolyte interphase during the formation cycles, the actual active area in the electrode is hypothesized to be a much smaller fraction of the theoretical area. Given that GITT based diffusivity and exchange current density computations are inversely proportional to S^2 and S respectively, it is likely that overestimation of area is leading to low diffusivity and exchange current density magnitudes obtained. Nevertheless, this does not affect the accuracy of qualitative trends of Si diffusivity and exchange current density variation with state of charge and comparison between Si and BSi.

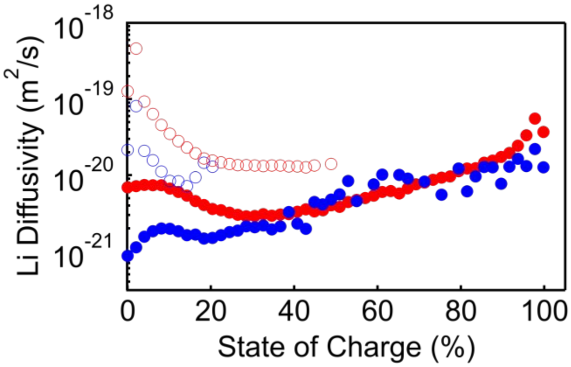

	Average Li Diffusivity	Exchange Current Density
BSi	7.6 x 10^{-20} m^2/s	1.2 x 10^{-4} A/m^2
Si	4.0 x 10^{-19} m^2/s	2.5 x 10^{-4} A/m^2

Figure S6: Lithium diffusivities as a function of the state of charge for the silicon (red) and BSi@Li_2CO_3 materials. The open shapes are the lithiation diffusivities and the closed shapes are the delithiation diffusivities.

Ion transport and electrode microstructure

Electrodes composed of very small NPs are expected to have a densely packed microstructure. At the electrode-level, mass transport can be quantified by the Macmillan number (N_M). N_M is the ratio of the ionic conductivity within the electrode compared to the ionic conductivity at a planar surface. Measuring the MacMillan number is performed under 'ion-blocking' conditions using $TBAPF_6$ electrolyte with a symmetric cell configuration. Under these conditions, the only contribution to capacitance is the electric double layer. Electrochemical impedance spectroscopy (EIS) was performed within a frequency range of 400 mHz-200 kHz. R_{ion}

– the effective ionic resistance –is determined by a linear extrapolation of the low-frequency line in the Nyquist plots Figure S7a and S7b. N_M was calculated using Equation 4.

$$N_M = \frac{\kappa}{\kappa_{eff}} = \frac{R_{Ion} \cdot A \cdot \kappa}{d}$$

Eq. 4

where, A is the electrode area, and d is the thickness of the electrode, and κ is the ionic conductivity of the electrolyte. The MacMullin number for the BSi@Li_2CO_3 anode is 29 and 18 for the pure silicon electrode.

To calculate the electrode tortuosity (τ), we use the empirical relationship[7]:

$$N_M = \frac{\tau}{\varepsilon}$$

Eq. 5

Where ε is the electrode porosity. To calculate the electrode porosity, we first measure the volume of the electrode by finding the average electrode thickness. Because the BSi electrodes have visible cracks, we estimate the volume % of the cracks by using the contrast between the cracks and the surface of the electrode and fitting the image with ImageJ software (Figure S7c and S7d). The crack volume accounts for ~6% of the total electrode volume. The crack volume is then subtracted from the measure volume to arrive at the electrode volume. The porosity is then calculated using the known composition and the component density. The densities used for this calculation were 0.16 g/cm^3 for the carbon nanotubes, 1.41g/cm^3 for the PI binder, and 2.33 g/cm^3 for the Si and BSi. Since the BSi@Li_2CO_3 and pure Si electrode are composed of particles with approximately the same size, the difference in N_M is the result of particle aggregation in the pure silicon electrodes which arises from a poorer dispersion in the slurry.

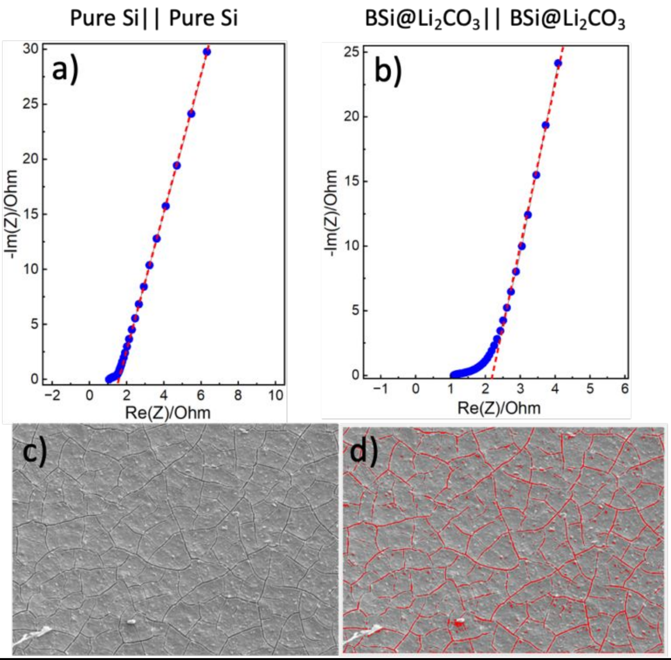

	Porosity (%)	MacMullin Number	Tortuosity (τ)
Pure Silicon	30	18	5.4
BSi anode	22	29	6.4

Figure S7: Nyquist plots for blocking electrolyte measurements of symmetric cells on (a) pure silicon electrodes and (b) BSi@Li_2CO_3. The electrolyte used in this experiment is 1.2M tetrabutyl ammonium hexafluorophosphate in acetonitrile. The red dashed line is a linear extrapolation to the intercept of the real axis. (c) and (d) show an SEM image of the BSi@Li_2CO_3 before and after deconvoluting the crack volume from the electrode volume. The crack volume is shown in red. The table below lists the parameters extracted from this measurement.

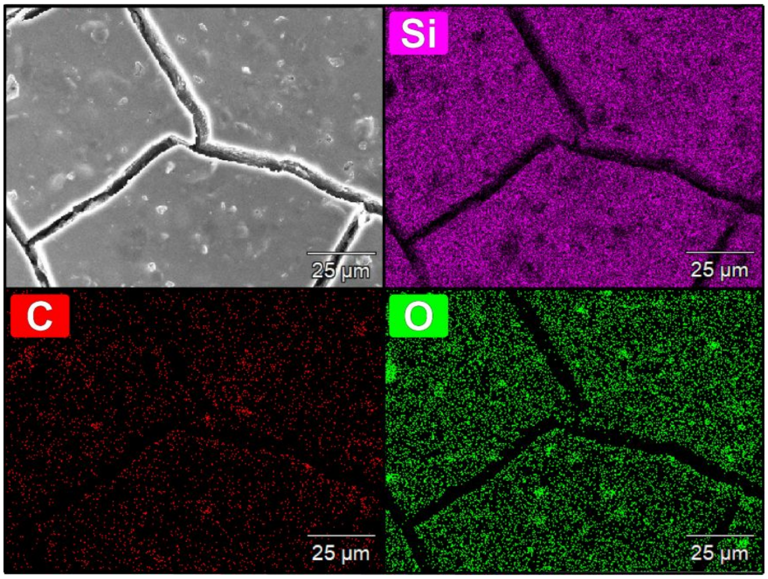

Figure S8. EDS spectra of a BSi@Li_2CO_3 NP composite anode before electrochemical cycling.

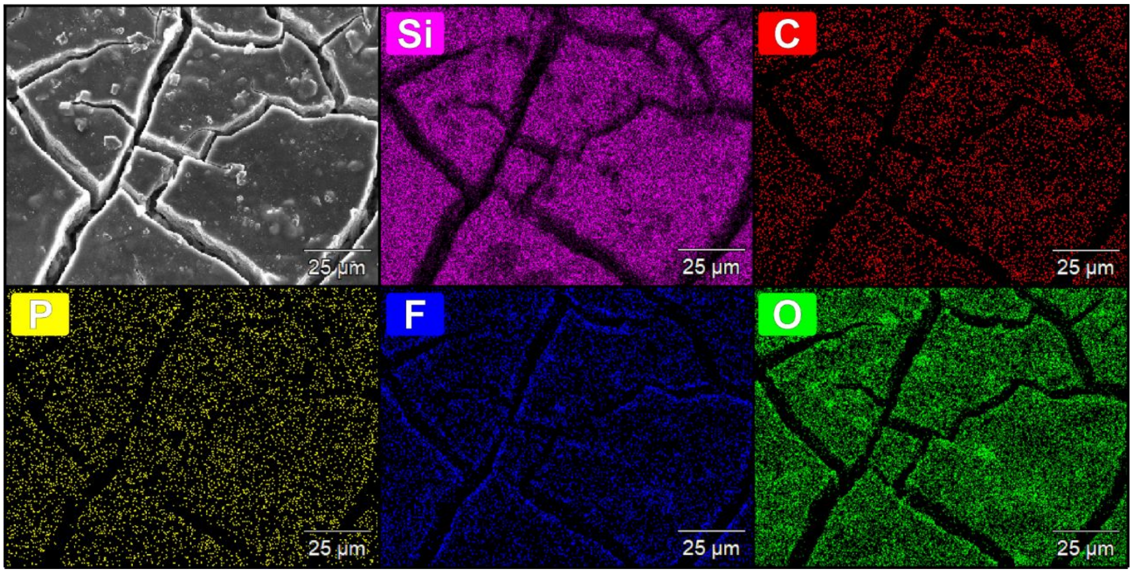

Figure S9. EDS spectra of a cycled BSi@Li_2CO_3 NP composite anode after 1000 cycles.

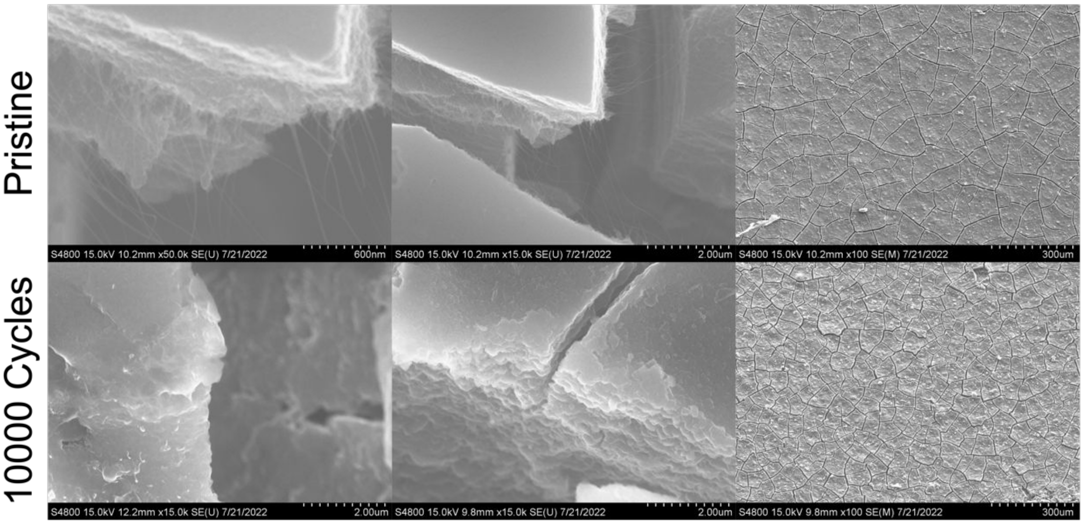

Figure S10: SEM images at various magnifications of pristine (top) and cycled (bottom) BSi@Li_2CO_3 NP composite anodes.

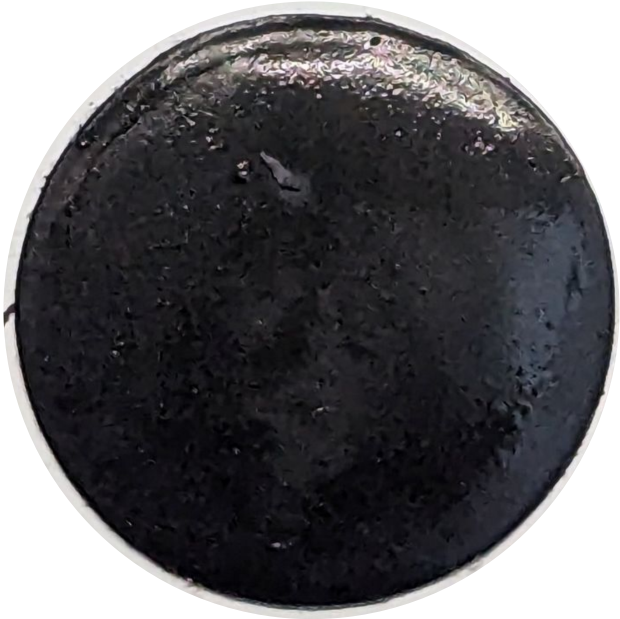

Figure S11: Unwashed BSi@Li_2CO_3 composite anode after electrochemical cycling.

References

(1) Schulze, M. C.; Urias, F.; Dutta, N. S.; Huey, Z.; Coyle, J.; Teeter, G.; Doeren, R.; Tremolet de Villers, B. J.; Han, S.-D.; Neale, N. R.; et al. Control of nanoparticle dispersion, SEI composition, and electrode morphology enables long cycle life in high silicon content nanoparticle-based composite anodes for lithium-ion batteries. *Journal of Materials Chemistry A* **2023**, *11* (10), 5257-5266, 10.1039/D2TA08935A. DOI: 10.1039/D2TA08935A.

(2) Wood, K. N.; Teeter, G. XPS on Li-Battery-Related Compounds: Analysis of Inorganic SEI Phases and a Methodology for Charge Correction. *ACS Applied Energy Materials* **2018**, *1* (9), 4493-4504. DOI: 10.1021/acsaem.8b00406.

(3) Verma, A.; Smith, K.; Santhanagopalan, S.; Abraham, D.; Yao, K. P.; Mukherjee, P. P. Galvanostatic Intermittent Titration and Performance Based Analysis of LiNi0.5Co0.2Mn0.3O2 Cathode. *J. Electrochem. Soc.* **2017**, *164* (13), A3380. DOI: 10.1149/2.1701713jes.

(4) Weppner, W.; Huggins, R. A. Determination of the Kinetic Parameters of Mixed-Conducting Electrodes and Application to the System Li3Sb. *J. Electrochem. Soc.* **1977**, *124* (10), 1569. DOI: 10.1149/1.2133112.

(5) Wang, M.; Xiao, X.; Huang, X. Study of lithium diffusivity in amorphous silicon via finite element analysis. *J. Power Sources* **2016**, *307*, 77-85. DOI: https://doi.org/10.1016/j.jpowsour.2015.12.082.

(6) Verma, A.; Franco, A. A.; Mukherjee, P. P. Mechanistic Elucidation of Si Particle Morphology on Electrode Performance. *J. Electrochem. Soc.* **2019**, *166* (15), A3852. DOI: 10.1149/2.0961915jes.

(7) Landesfeind, J.; Hattendorff, J.; Ehrl, A.; Wall, W. A.; Gasteiger, H. A. Tortuosity Determination of Battery Electrodes and Separators by Impedance Spectroscopy. *J. Electrochem. Soc.* **2016**, *163* (7), A1373. DOI: 10.1149/2.1141607jes.
